# Videofluoroscopic Evaluation of the Pharynx and Upper Esophageal Sphincter in the Dog: A Systematic Review of the Literature

**DOI:** 10.3389/fvets.2019.00117

**Published:** 2019-04-24

**Authors:** Rachel E. Pollard

**Affiliations:** Department of Surgical and Radiological Sciences, School of Veterinary Medicine, University of California, Davis, Davis, CA, United States

**Keywords:** videofluroscopy, pharynx, upper esophageal sphincter (UES), dysphagia, pharyngeal collapse

## Abstract

**Background:** Diseases of the pharynx and upper esophageal sphincter can result in debilitating respiratory difficulty, dysphagia or a combination of both. An exact diagnosis is essential to properly prognosticate and guide therapy. Videofluoroscopic assessment of the pharynx and upper esophageal sphincter with or without orally administered contrast material is the diagnostic of choice for many diseases as both anatomic and functional information is gleaned. The purpose of this review is to assess for continuity in imaging protocols across institutions and to record quantitative and qualitative parameters used for analysis of videofluoroscopy of the pharynx and upper esophageal sphincter in dogs.

**Methods:** A systematic literature search was performed including articles published in peer-reviewed veterinary journals involving the topic of videofluoroscopy of the pharynx and upper esophageal sphincter through August 1, 2018. Specifics of study acquisition technique were recorded. Quantitative and qualitative videofluoroscopic parameters were recorded and compared across institutions where appropriate using one-way ANOVA with *p* ≤ 0.05 being considered significant.

**Results:** Videofluoroscopy of the pharynx and upper esophageal sphincter is performed either in right lateral or standing postures depending on the institution. Bolus size and consistency used during contrast videofluoroscopy of swallowing differs between institutions. Some institutions evaluate videofluoroscopic studies using qualitative criteria while others apply quantitative measures. Reported quantitative measures include inter-swallow interval, swallow rate, jaw cycles per swallow ratio, time to upper esophageal opening, maximal pharyngeal contraction, maximum laryngeal excursion, upper esophageal closure, epiglottic re-opening, and pharyngeal constriction ratio. Measurement outcomes are significantly different between institutions and when bolus size/consistency is variable when assessing healthy dogs.

**Conclusions:** The current peer-reviewed literature on fluoroscopic evaluation of the pharynx and UES in dogs shows a lack of standardization regarding imaging protocol. There is not a standard set of quantitative criteria applied amongst the institutions and there are significant differences in the outcomes obtained from videofluoroscopic assessment of swallowing suggesting significant inter-observer or inter-institutional variability. A consensus statement regarding imaging protocol and what parameters should be used to interpret airway and swallowing videofluoroscopic studies of the pharynx and UES in dogs is needed along with targeted analysis of observer variability.

## Introduction

### Rationale

Diseases of the pharynx and upper esophageal sphincter can result in debilitating respiratory difficulty, dysphagia or a combination of both. Dysphagia is defined as difficulty swallowing as a result of disturbance of the oral, pharyngeal or esophageal phases of swallowing (1). The oral phase of swallowing is voluntary and results in transport of a bolus to a midline position between the tongue base and hard palate. The pharyngeal phase is the involuntary transport of the bolus past the pharynx and through the upper esophageal sphincter into the proximal esophagus. The esophageal phase transports the bolus from the proximal esophagus through the lower esophageal sphincter to the stomach (1). For the purposes of this review, focus will be placed on assessment of dogs with respiratory difficulty or pharyngeal dysphagia resulting from anatomic or functional disorders of the pharynx and upper esophageal sphincter.

The pharynx can be sub-divided into the nasopharynx (area dorsal to the soft palate between the choanae and the intrapharyngeal opening), oropharynx (area ventral to the soft palate between the palatoglossal arches and the epiglottis) and the laryngopharynx (the most caudal part of the pharynx) (1). In the dog, there are a variety of anatomic abnormalities which can affect the pharynx which include foreign bodies, stenoses, abscesses, tumors, polyps, and cysts derived from Rathke's pouch or the nasophayngeal mucosa (2–6). Thickening and elongation of the soft palate or the presence of nasopharyngeal sialoceles are causes of pharyngeal dysfunction most commonly occurring in brachycephalic breeds (7, 8). Functional abnormalities of the pharynx typically result from primary (myasthenia gravis, cranial nerve dysfunction, rabies) or secondary (pharyngeal collapse resulting from long-term negative pressure gradients from airway obstruction) causes of neuromuscular weakness (9, 10).

Diseases of the upper esophageal sphincter (UES) may or may not accompany abnormalities of the pharynx. Anatomic abnormalities that may affect the UES include foreign bodies and strictures while the most common functional abnormalities include cricopharyngeal dyssynchrony and cricopharyngeal achalasia. Cricopharyngeal dyssynchrony is a congenital or acquired disorder which involves delayed opening of the UES relative to bolus presentation (11). Cricopharyngeal achalasia is most commonly congenital and refers to incomplete or absent opening of the UES (10, 12). Achalasia and dyssynchrony may occur simultaneously (13). With pharyngeal, UES and combined pharyngeal-UES dysfunction, an exact diagnosis of the underlying problem is essential to properly prognosticate and guide therapy (14).

Videofluoroscopic assessment of the pharynx and upper esophageal sphincter with or without orally administered contrast material is the diagnostic of choice for many diseases as both anatomic and functional information is gleaned (10, 12, 15). The presence of severe anatomic abnormalities or transient events such as pharyngeal collapse may be visible using videofluoroscopy without oral contrast material. However, functional and subtle anatomic abnormalities, particularly those which result in dysphagia, often require videofluoroscopic observation of swallowing during oral administration of barium and barium soaked food to determine the underlying cause. To the authors knowledge, there is no continuity regarding how videofluoroscopic swallowing studies (VFSS) are performed across institutions nor are there a standard set of quantitative and qualitative parameters that are evaluated.

### Objectives

The purpose of this review is to determine the current videofluoroscopy protocols used by the various institutions which are publishing data about the pharynx and UES of dogs. In addition, quantitative, semi-quantitative and qualitative parameters used for analysis of videofluoroscopy of the pharynx and upper esophageal sphincter were compiled and compared across institutions. The overarching goal of this review is to assess whether improved standardization of videofluoroscopic study acquisition and analysis is warranted.

## Materials and Methods

### Study Design

A systematic review of the current literature was performed, including articles published between August 1, 2008 and August 1, 2018, for manuscripts relating to videofluoroscopy of the pharynx and UES in dogs. PubMed was searched using the following terms: dog or canine and pharynx or upper esophageal sphincter or cricopharyngeal or swallowing or dysphagia and videofluoroscopy or fluoroscopy or esophagram. Inclusion and exclusion criteria were predetermined so as to minimize bias. Articles from peer-reviewed journals were included if data presented was original (i.e., review articles were excluded). The institution from which the publication originated, the type of study design (prospective or retrospective), and the study population (healthy or clinically ill dogs) was recorded. Specifics of videofluoroscopy protocol (patient positioning, use of a restraint device, frame rate of image acquisition, types of contrast material and food administered) were collected. Quantitative, semi-quantitative, and qualitative mechanisms for evaluating the pharynx and UES were recoded including standards for normal and abnormal interpretation. Case reports were eligible for inclusion if the imaging protocol and criteria applied to diagnosis were clearly outlined.

### Statistical Analysis

Under consultation with a statistician, a one-way ANOVA from summary data was performed to compare the quantitative results obtained from studies performed on healthy dogs to determine if results were comparable (Statpages.info/anova1sm.html; freeware). A Tukey HSD (Honesty Significant Difference) *post-hoc* evaluation was also performed to determine which specific data from individual studies were significantly different from one another. *P* ≤ 0.05 was considered significant.

## Results

**Figure 1:**
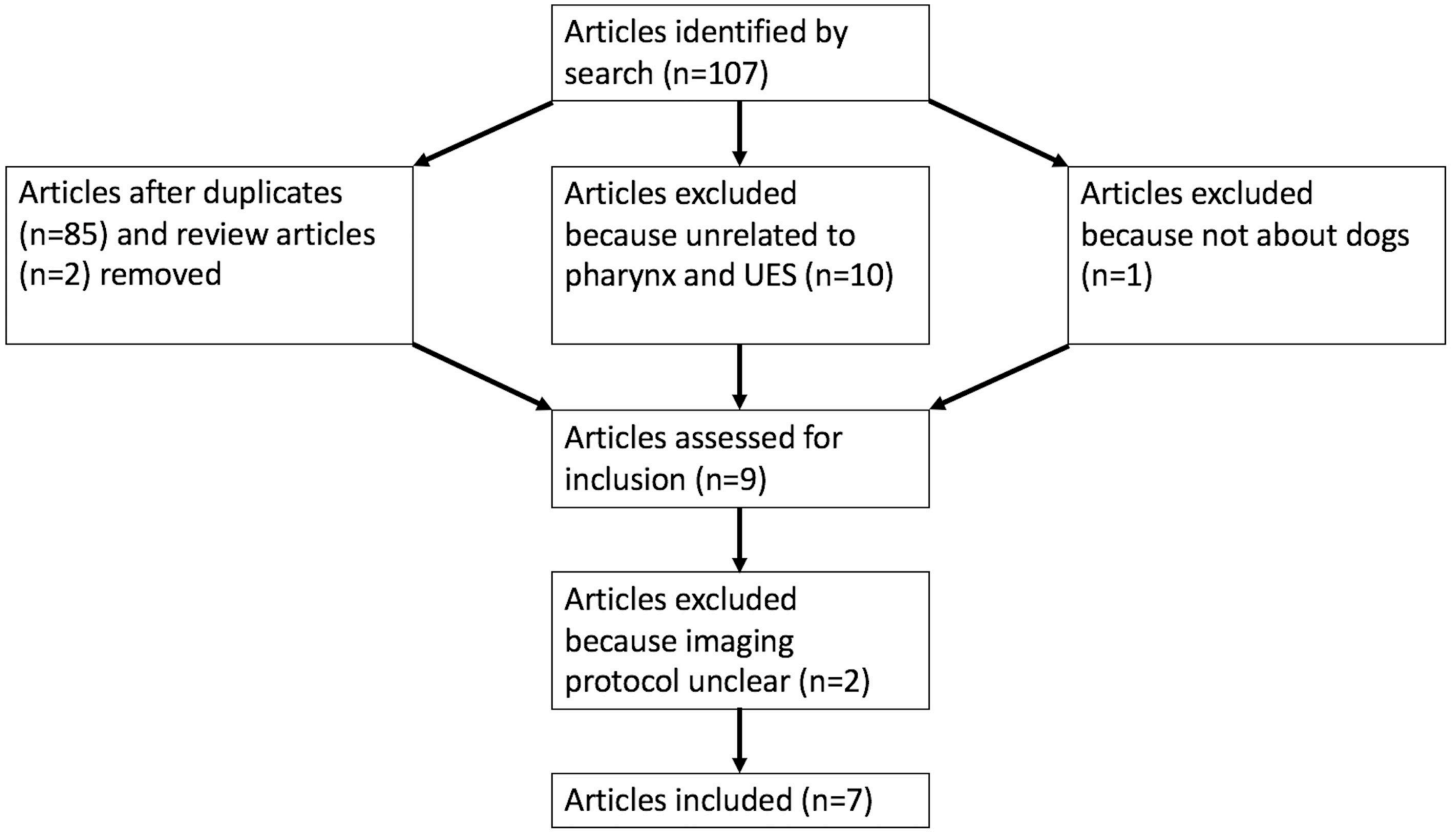
A flowchart is presented demonstrating the process by which articles were selected for inclusion (UES, upper esophageal sphincter).

PubMed search yielded 107 potential articles for inclusion. A flowchart (Figure 1) in included showing the process by which articles were included or eliminated with a total of 7 articles ultimately meeting the inclusion criteria (9, 13, 15–19). The specifics of institution of origin, type of study, and study population are shown in Table 1.

**Table 1:** A list of the 7 studies included in the study is included along with the institution from which the work originated, study design and population of dogs included in the study.

**Study**	**Institution**	**Study design**	**Study population**
Andrade et al. (16)	University of Georgia	Prospective	Dogs with laryngeal paralysis
Bonadio et al. (17)	University of California, Davis	Prospective	Healthy dogs
Cheney et al. (18)	University of California, Davis	Prospective	Healthy dogs
Harris et al. (19)	University of Missouri	Prospective	Healthy dogs
Pollard et al. (13)	University of California, Davis	Retrospective	Dogs with dysphagia
Pollard et al. (15)	University of California, Davis	Retrospective	Dogs undergoing airway or swallowing fluoroscopy
Rubin et al. (9)	University of Pennsylvania	Retrospective	Dogs undergoing airway fluoroscopy

Considering the seven included studies (4 performed at 1 institution and 1 each at 3 other institutions), four reported that image acquisition was performed with the dogs positioned in right lateral recumbency (13, 15, 16, 18), one reported that image acquisition was performed with the dogs standing (19), one reported that image acquisition was performed with dogs positioned in both right lateral and standing with the intention of determining the impact of body position (17) and one reported that image acquisition was performed with dogs in either right lateral or standing depending on demeanor and clinical stability (9). Positioning devices were used in 3 studies to allow for standing/sternal posture. Harris et al. custom built polycarbonate kennels of 4 differing sizes (small/toy <3.2 to ≤16 kg; medium >16 to ≤29 kg; large >29 to ≤39 kg; giant ≥39 kg) to accommodate free feeding for a variety of different dog sizes (19). Kennels were funnel (trapezoidal) shaped with an adjustable ring at one end in which to place the food bowl for free feeding. Bonadio et al. reported the use of a custom polycarbonate kennel with open ends to allow feeding and an adjustable long side to accommodate differing dog widths (17). Rubin et al. reported restraining dogs in a plastic box for fluoroscopic evaluation with no further details provided (9). The frame rate of image acquisition was reported as 30 frames per second for 5 studies (13, 15, 17–19) while two studies did not report the frame rate (9, 16).

Two of the 7 studies were specifically looking for pharyngeal collapse using fluoroscopy during tidal respiration (9, 15). Both were retrospective studies and defined pharyngeal collapse semi-quantitatively as complete if the entire pharyngeal lumen was lost or partial if luminal diameter was reduced by >50% but <100%. Fluoroscopy was being used for airway assessment in the first of these two studies so that no oral contrast media was administered (9). Twenty-eight dogs diagnosed with pharyngeal collapse were evaluated in this study, twenty-seven of which had one or more types of concurrent cardiorespiratory disease (most commonly tracheal collapse *n* = 17; mainstem bronchial collapse *n* = 18; brachycephalic airway syndrome *n* = 8). Twenty of 28 dogs had complete pharyngeal collapse. No other imaging parameters were assessed. The patient population in the second study included dogs undergoing airway or swallowing videofluoroscopy but analyzed only the portion of the fluoroscopic study during tidal respiration so that no swallowing parameters were reported (15). Results of this study indicated that pharyngeal collapse was more common in brachycephalic breeds (72%) compared to dolichocephalic/mesocephalic dogs with (28%) or without (7%) airway collapse.

Five of the 7 studies were evaluating the pharynx and UES using contrast videofluoroscopy with the intent of quantifying swallowing parameters that would be relevant to dogs with dysphagia (13, 16–19). The consistencies and sizes of boluses administered are listed in Table 2. Three of the 5 studies were performed imaging healthy dogs to create reference ranges for comparison to dogs with dysphagia (17–19). One study compared the outcome of quantitative measures applied to healthy dogs when imaged in right lateral recumbency to those from the same dogs when imaged in sternal recumbency and found that quantitative pharyngeal and UES measurements did not significantly differ with body position (17). Another study evaluated the impact of bolus size on quantitative measures of pharyngeal and UES function in healthy dogs and concluded that bolus sizes should be standardized to minimize variability (18). The third study developed a standardized protocol for VFSS and compared quantitative measures of pharyngeal and UES function between healthy juvenile (0.1 ± 0.6 years), adult (4.9 ± 0.93 years) and geriatric (11.3 ± 3.55 years) dogs finding no significant difference between age groups (19).

**Table 2:** A summary of the types, sizes and numbers of liquid, soft food, and kibble boluses administered during the five studies in which videofluoroscopic swallowing studies were performed is provided.

**Study**	**Liquid bolus**			**Soft food bolus**			**Kibble bolus**		
	**Type**	**Size**	**Number**	**Type**	**Size**	**Number**	**Type**	**Size**	**Number**
Andrade et al. (16)	Barium; E-Z-Paque	12 ml total	≥5	Hills a/d mixed with 5 ml barium	$\frac{1}{2}$ can total	≥5	NA	NA	NA
Bonadio et al. (17)	Barium; Novopaque	5–10 ml per bolus	3–5	NA	NA	NA	NR	5–10 kibbles per bolus	3–5
Cheney et al. (18)	Barium; Novopaque	5, 10, 15 ml per bolus	≥3 of each size	Purina ProPlan	3, 8, 12 gr	≥3 of each size	NA	NA	NA
Harris et al. (19)	25% Iohexol (350 mg Iodine/ml) diluted with chicken broth	Free fed	≥3	Pureed canned food mixed with 25% Iohexol	Free fed	≥3	Kibble mixed with 40% barium powder	Free fed	≥3
Pollard et al. (13)	Barium; Novopaque	3–5 ml per bolus	3–5	NA	NA	NA	NR	5–6 kibbles per bolus	3–5

*NA, not assessed; NR, not reported*.

**Figure 2:**
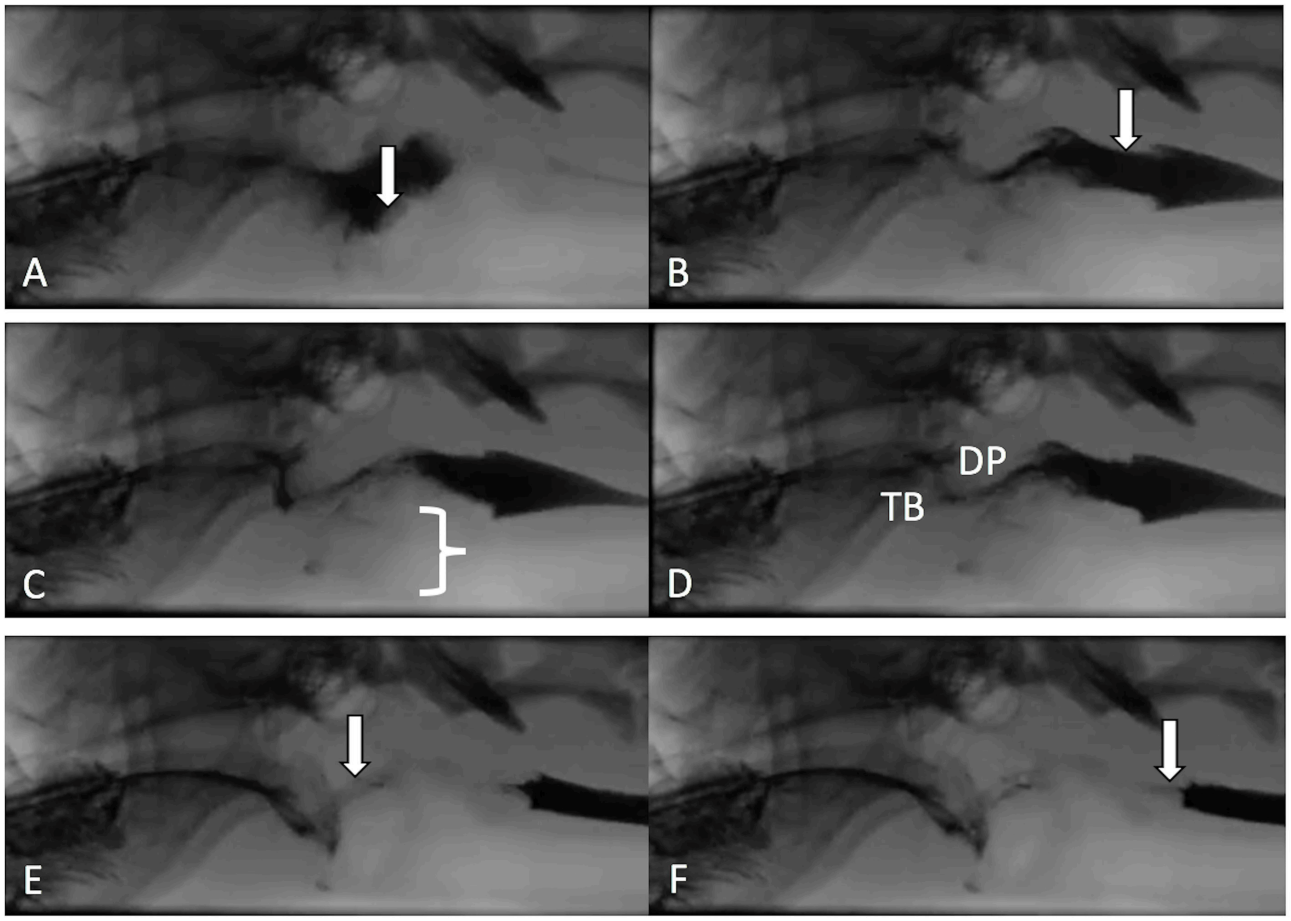
Representative images are shown from a contrast videofluoroscopic evaluation of swallowing obtained from a healthy dog. Typically, the number of frames is counted from onset of swallowing to specified events and converted to seconds based on 30 frame per second image acquisition. **(A)** Swallow onset frame indicated by closure of the epiglottis (white arrow) and perpendicular movement of the bolus toward the pharynx. **(B)** First frame in which the upper/proximal esophageal sphincter (white arrow) is open. **(C)** Frame in which the larynx is in its most rostral position (white bracket). **(D)** Frame obtained at maximal pharyngeal contraction where tongue base (TB) and dorsal pharyngeal wall (DP) are in contact and have their most ventral and caudal position. **(E)** Frame in which the air column in the pharynx has reappeared (white arrow) due to opening of the epiglottis and relaxation of the pharynx. **(F)** Frame in which the bolus has completely passed into the proximal esophagus and the upper/proximal esophageal sphincter is closed (white arrow).

**Figure 3:**
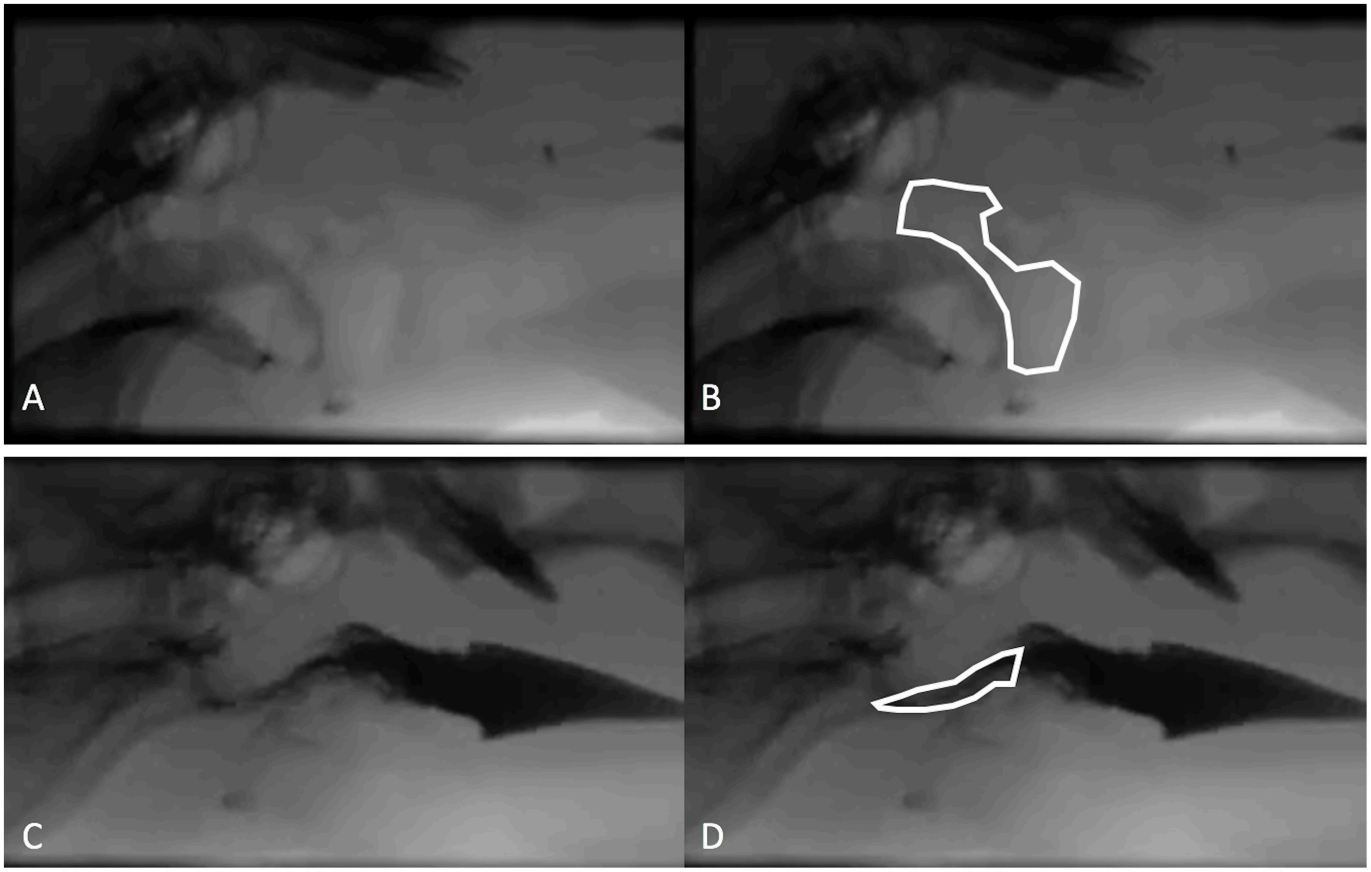
The pharyngeal constriction ratio (PCR) is a metric of pharyngeal function and is calculated by measuring the area in the pharynx at maximal contraction (maximal contraction frame) and dividing that by the area of the pharynx at rest (hold frame). **(A)** Hold frame from a contrast videofluoroscopic evaluation of swallowing obtained from a healthy dog. **(B)** Same image as in A with the area of pharynx outlined including the air space beginning dorsal to the soft palate then rostrally to the hyoid apparatus and tympanic bulla, dorsally to the dorsal aspect of the pharyngeal wall, caudally along the dorsal aspect of the pharyngeal wall to the cranial esophageal sphincter, ventrally around the corniculate process of the arytenoid cartilage to include the vallecula, finally connecting the epiglottis to the starting point. **(C)** Maximal contraction frame from the same dog as in **(A,B)**. **(D)** Maximal contraction frame from the same dog as in **(A,B)** with the area of the pharynx defined.

The quantitative parameters that were measured varied between studies and included the inter-swallow interval (ISI; time in seconds between 2 successive, uninterrupted swallows from the onset frame of swallow 1 to the onset frame of the subsequent swallow), swallow rate (the number of swallows per 3-second interval of uninterrupted prehension), jaw cycles per swallow ratio (the number of licks from maximum jaw excursion to the following maximum jaw excursion), time from swallow onset (Figure 2A) to proximal/upper esophageal sphincter opening (Figure 2B), time to maximum laryngeal excursion (Figure 2C), time to maximum pharyngeal contraction (Figure 2D), time to air column reopening/epiglottis re-opening (Figure 2E), time to proximal/upper esophageal sphincter closure (Figure 2F), and pharyngeal constriction ratio (PCR; Figures 3A–D) (19–21). Tables 3–5 outline the quantitative parameters reported, the ranges established and the statistical comparisons between data obtained by these 3 studies.

**Table 3:** Presented is a summary of three studies evaluating quantitative parameters obtained during videofluoroscopic assessment of swallowing of liquid boluses in healthy dogs.

	**Bonadio et al**. (17)		**Cheney et al**. (18)			**Harris et al**. (19)		
**Parameter**	**Lateral**	**Standing**	**Small bolus**	**Medium bolus**	**Large bolus**	**Juvenile**	**Adult**	**Geriatric**
UES open (s) (*p* = 0.000)	0.10 ± 0.02^@^	0.11 ± 0.02^#^	0.09 ± 0.02^%∧^	0.08 ± 0.02^#^	0.08 ± 0.02^#&^	0.04 ± 0.02^@#%∧&^	0.05 ± 0.03^@#%∧&^	0.03 ± 0.02^@#%∧&^
Max laryngeal excursion (s)	NA	NA	NA	NA	NA	0.07 ± 0.03	0.08 ± 0.04	0.10 ± 0.06
Max pharyngeal contraction (s) (*p* = 0.0001)	0.11 ± 0.04^@^	0.10 ± 0.03^#^	0.15 ± 0.04^#^	0.16 ± 0.04^@#^	0.17 ± 0.04^@#%^	0.11 ± 0.06	0.12 ± 0.04	0.11 ± 0.02^%^
Epiglottis open (s) (*p* = 0.025)	0.24 ± 0.04	0.25 ± 0.05	0.22 ± 0.05^@^	0.23 ± 0.04	0.25 ± 0.04	0.28 ± 0.03	0.26 ± 0.06	0.30 ± 0.05^@^
UES closed (s) (*p* = 0.0001)	0.25 ± 0.04^@^	0.27 ± 0.04^#^	0.25 ± 0.06^%^	0.26 ± 0.05^∧^	0.29 ± 0.05	0.33 ± 0.03^@^	0.31 ± 0.06^@^	0.35 ± 0.06^@#%∧^
PCR (*p* = 0.99)	0.11 ± 0.35	0.14 ± 0.04	0.13 ± 0.03	0.13 ± 0.03	0.13 ± 0.04	NA	NA	NA
ISI (s)	NA	NA	NA	NA	NA	1.51 ± 1.97	1.14 ± 0.53	1.19 ± 0.69
Swallow rate (per 3 s)	NA	NA	NA	NA	NA	1.65 ± 1.20	2.65 ± 0.70	2.35 ± 0.65
Jaw cycles per swallow ratio	NA	NA	NA	NA	NA	2.70 ± 2.68	2.85 ± 1.15	3.00 ± 1.25

*Bonadio et al. (17) compared results when dogs were positioned in right lateral vs. standing body position. Cheney et al. (18) compared results when dogs were administered different bolus sizes. Harris et al. (19) compared results obtained from dogs of differing ages [juvenile (0.1 ± 0.6 years), adult (4.9 ± 0.93 years), geriatric (11.3 ± 3.55 years)]. One-way ANOVA was performed to evaluate for statistically significantly different results across studies. A Tukey HSD (Honesty Significant Difference) post-hoc evaluation was also performed to determine which specific data were significantly different from one another. P ≤ 0.05 was considered significant. (^@#%∧&^denotes significant differences between values in different columns but in the same row)*.

*NA, not assessed; UES, upper esophageal sphincter; s, seconds; Max, maximum; PCR, pharyngeal constriction ratio; ISI, inter-swallow interval*.

**Table 4:** Presented is a summary of 2 studies evaluating quantitative parameters obtained during videofluoroscopic assessment of swallowing of soft food boluses in healthy dogs.

	**Cheney et al**. (18)			**Harris et al**. (19)		
**Parameter**	**Small bolus**	**Medium bolus**	**Large bolus**	**Juvenile**	**Adult**	**Geriatric**
UES open (s) (*p* = 0.0001)	0.10 ± 0.02^@^	0.09 ± 0.02^%^	0.08 ± 0.02^∧^	0.03 ± 0.02^@%∧&^	0.06 ± 0.03^@^	0.09 ± 0.04^&^
Max laryngeal excursion (s)	NA	NA	NA	0.07 ± 0.02	0.14 ± 0.04	0.09 ± 0.03
Max pharyngeal contraction (s) (*p* = 0.000)	0.17 ± 0.02^@^	0.19 ± 0.03^∧^	0.19 ± 0.02^#^	0.11 ± 0.03^@∧#^	0.12 ± 0.02^@∧#^	0.12 ± 0.02^@∧#^
Epiglottis open (s) (*p* = 0.64)	0.26 ± 0.07	0.26 ± 0.03	0.27 ± 0.03	0.27 ± 0.04	0.30 ± 0.08	0.28 ± 0.08
UES closed (s) (*p* = 0.000)	0.28 ± 0.05^@^	0.30 ± 0.05^#^	0.30 ± 0.06^%^	0.34 ± 0.02	0.39 ± 0.05^@#%^	0.37 ± 0.05^@#^
PCR (*p* = 0.000)	0.11 ± 0.03^@^	0.12 ± 0.02^#^	0.12 ± 0.02^%^	0.04 ± 0.01^@#%^	0.05 ± 0.04^@#%^	0.02 ± 0.01^@#%^
ISI (s)	NA	NA	NA	2.53 ± 0.40	3.14 ± 1.49	4.04 ± 1.75
Swallow rate (per 3 s)	NA	NA	NA	2.00 ± 0.08	1.50 ± 1.00	1.25 ± 0.65
Jaw cycles per swallow ratio	NA	NA	NA	7.7 ± 2.3	8.0 ± 5.7	9.35 ± 5.68

*Cheney et al. (18) compared results when dogs were administered different bolus sizes. Harris et al. (19) compared results obtained from dogs of differing ages [juvenile (0.1 ± 0.6 years), adult (4.9 ± 0.93 years), geriatric (11.3 ± 3.55 years)]. One-way ANOVA was performed to evaluate for statistically significantly different results across studies. A Tukey HSD (Honesty Significant Difference) post-hoc evaluation was also performed to determine which specific data were significantly different from one another. P ≤ 0.05 was considered significant. (^@#%∧&^denotes significant differences between values in different columns but in the same row)*.

*NA, not assessed; UES, upper esophageal sphincter; s, seconds; Max, maximum; PCR, pharyngeal constriction ratio; ISI, inter-swallow interval*.

**Table 5:** Presented is a summary of 2 studies evaluating quantitative parameters obtained during videofluoroscopic assessment of swallowing of kibble boluses in healthy dogs.

	**Bonadio et al**. (17)		**Harris et al**. (19)		
**Parameter**	**Lateral**	**Standing**	**Juvenile**	**Adult**	**Geriatric**
UES open (s) (*p* = 0.18)	0.14 ± 0.06	0.15 ± 0.05	0.08 ± 0.05	0.12 ± 0.11	0.09 ± 0.04
Max laryngeal excursion (s)	NA	NA	0.14 ± 0.12	0.24 ± 0.05	0.14 ± 0.12
Max pharyngeal contraction (s)	0.13 ± 0.06	0.14 ± 0.04	NA	NA	NA
Epiglottis open (s) (*p* = 0.000)	0.29 ± 0.06^@^	0.26 ± 0.04^#^	0.37 ± 0.17	0.56 ± 0.27^@#^	0.53 ± 0.10^@#^
UES closed (s) (*p* = 0.000)	0.33 ± 0.07^@^	0.28 ± 0.05^#^	0.43 ± 0.44^%^	1.02 ± 0.62^@#%^	0.65 ± 0.12
PCR	0.11 ± 0.04	0.11 ± 0.21	NA	NA	NA
ISI (s)	NA	NA	6.61 ± 3.44	5.25 ± 2.90	6.43 ± 3.65
Swallow rate (per 3 s)	NA	NA	1.25 ± 0.63	1.30 ± 0.70	1.00 ± 0
Jaw cycles per swallow ratio	NA	NA	15.5 ± 6.5	12.0 ± 9.3	10.0 ± 4.5

*Bonadio et al. (17) compared results when dogs were positioned in right lateral vs. standing body position. Harris et al. (19) compared results obtained from dogs of differing ages [juvenile (0.1 ± 0.6 years), adult (4.9 ± 0.93 years), geriatric (11.3 ± 3.55 years)]. One-way ANOVA was performed to evaluate for statistically significantly different results across studies. A Tukey HSD (Honesty Significant Difference) post-hoc evaluation was also performed to determine which specific data were significantly different from one another. P ≤ 0.05 was considered significant. (^@#%^denotes significant differences between values in different columns but in the same row)*.

*NA, not assessed; UES, upper esophageal sphincter; s, seconds; Max, maximum; PCR, pharyngeal constriction ratio; ISI, inter-swallow interval*.

The remaining 2 studies reported the outcome of contrast videofluoroscopic swallowing studies performed on dogs with laryngeal paralysis (16) or dysphagia (13). Andrade et al. performed contrast videofluoroscopic assessment of swallowing in dogs with idiopathic laryngeal paralysis before and after unilateral arytenoid lateralization. Pharyngeal and UES parameters assessed were primarily qualitative and included presence or absence of tracheal contamination with contrast material and subjective assessment of motility and coordination of the pharynx. Only one dog was reported to have delayed pharyngeal emptying prior to surgery and there was no follow-up imaging performed so that improvement could not be assessed (16). Pollard et al. retrospectively reported the outcome of contrast videofluoroscopy of swallowing in 216 dogs with dysphagia (13). In this study, the quantitative parameters used to evaluate the pharynx and UES included time to upper esophageal sphincter opening, time to maximum pharyngeal contraction, time to epiglottis re-opening, time to upper esophageal sphincter closure, and PCR. Twenty-seven dogs (13%) in this study were diagnosed with pharyngeal weakness based on elevation of pharyngeal constriction ratio (mean ± SD = 0.7 ± 0.4) with normal timing of UES opening (mean ± SD = 0.09 ± 0.06 sec). Seventeen dogs (8%) were diagnosed with cricopharyngeal disease; 6 with asynchrony based on delayed UES opening relative to timing of maximum pharyngeal contraction (mean ± SD = 0.16 ± 0.06), 6 with achalasia based on absent or insufficient UES opening and 5 with both based on the combination of delayed and insufficient UES opening. Dogs with cricopharyngeal origin dysphagia had elevation of pharyngeal constriction ratio due to the obstructive nature of the closed UES (mean ± SD = 0.8 ± 0.7). Overall results of this study indicated that 38% of dogs had abnormalities affecting more than one phase of swallowing.

## Discussion

### Summary of Main Findings

The results of this study indicate that, while most institutions report image acquisition to occur at 30 frames per second when performing airway and swallowing fluoroscopy, there is a lack of standardization with regards to general fluoroscopy protocols across differing institutions. More specifically, in the 2 studies which explicitly evaluated the pharynx for the presence or absence of collapse, one institution obtained fluoroscopic images with the dog positioned in right lateral recumbency while the other institution obtained fluoroscopic images with the dog either in right lateral or in a standing position (9, 15). There is a lack of literature comparing the impact of body position on the ability to rightfully or wrongfully visualize pharyngeal collapse or to characterize collapse as partial or complete so that comparing the results of these 2 studies is difficult. Regardless, the results of these 2 retrospective studies indicate that pharyngeal collapse is common in brachycephalic breeds and dogs with airway collapse with most dogs have complete rather than partial collapse.

Disparity was also seen with imaging protocols in the 5 studies reporting VFSS (13, 16–19). Three of these studies were performed on healthy dogs with the intention of either establishing a standard protocol (19) or clarifying what inconsistencies would have a significant impact on study outcome (17, 18). Body position was variable between institutions with some performing VFSS with the dogs positioned in right lateral recumbency and some standing in an adjustable “box” to emulate a natural eating posture. At least one study evaluated the impact of body position on quantitative measures of swallowing and found that esophageal transit was significantly impacted but that quantitative assessments of the pharyngeal and cricopharyngeal swallowing phases were not (17). As such, quantitative results gleened from studies performed in differing body positions and specific to the pharynx and UES should be comparable. Nevertheless, the types and sizes of liquid contrast material and food boluses were highly variable between institutions and one study indicated that the size and consistency of the bolus did have a significant impact on quantitative parameters (18). These findings are in agreement with previous literature indicating that larger bolus sizes result in earlier opening and later closure of the UES so as to biomechanically accommodate the increase in liquid or food volume (22).

Review of the literature and personal consultation with a specialist in otolaryngology indicates that there is no consensus regarding a protocol for performing VFSS in people with dysphagia (personal communication with Peter Belafsky MD, PhD, Medical Director of the Voice and Swallowing Center University of California, Davis). Unalike protocols are reported by different institutions in the United States currently publishing in this area of research (23–25) although protocol consensus may be consistent loco-regionally or in different countries. The method for assessment of VFSS studies is also not standardized in people despite the fact that a comprehensive set of quantitative measures have been developed, reported and validated (26). Qualitative VFSS assessment has proven inconsistent relative to quantitative assessment so that qualitative evaluation correctly classifies VFSS studies as normal or abnormal only 61.5% of the time (27). Intra-rater (*K* = 0.43–0.83) and inter-rater (*K* = 0.40–0.59) agreement was highly variable and evaluators agreed on a correct interpretation only 28% of the time (27). Rater experience is another factor which has proven impactful on the outcome of VFSS assessment in people with reviewers who are trained to use quantitative evaluation tools having significantly higher accuracy, inter- and intra-rater reliability (28).

One might presume that inter-observer variability might be even greater in veterinary patients where the body conformation of the subjects is radically different from breed to breed so that selecting the correct anatomical landmarks may be more challenging. The statistical comparison of quantitative parameters reported from different institutions in our study would support inter-observer or potentially inter-institutional variability as having a significant impact on outcome. More specifically, the majority of the results which were significantly different when comparing values obtained from healthy dogs were time to UES opening, time to maximal pharyngeal contraction, time to UES closed and PCR calculated at 2 different institutions (17–19). The values that were calculated in different studies performed at the same institution were not nearly as likely to be significantly different and, when different, were attributable to differing bolus size (17, 18). This outcome would indicate that inter-observer or inter-institutional variability is likely to impact the mechanics by which calculations are performed. Observer experience likely also plays a role in both quantitative and qualitative study interpretation. Alternatively, the regional differences between the population of dog breeds available to participate in research studies might impact averaged data based on the fact that perhaps an overarching reference range for all dog breeds regardless of size or cephalic anatomy is inappropriate. A study comparing observer experience, inter-observer and inter-institutional variability for the assessment of quantitative swallowing parameters would be necessary to further determine whether comparison of results between raters and institutions is reliable.

### Limitations

Several limitations must be considered with reference to the results presented in this systematic review. The implementation of pre-determined inclusion and exclusion criteria was intended to eliminate bias but resulted in at least 2 case report studies being eliminated based on unclear imaging protocol definition (29, 30). Had those studies been more descriptive so that they were included, perhaps a more consistent pattern for videofluoroscopic protocol across institutions may have emerged. Moreover, both of those studies used qualitative criteria to make the diagnosis so that an institutional pattern of qualitative vs. quantitative VFSS evaluation may have become apparent. As reported, the results indicate that most institutions use quantitative methods of VFSS evaluation. However, the data is highly skewed toward the protocol of one institution from which 4 of the studies originated.

An additional limitation to this review is the small number of studies published on the topics of pharyngeal and UES fluoroscopy in the last 10 years. Had a longer timeframe been included, additional publications may have come to light and influenced the results. The 10-year timeline was chosen so as to focus on what is the most current status of videofluoroscopic image acquisition and analysis in dogs but it is clear that this area of investigation is limited.

Finally, there is a paucity of information regarding the impact of quantitative vs. qualitative assessment of VFSS performed in dogs on study outcome and patient treatment. Extrapolation of experience from VFSS evaluation in people suggests that quantitative outperformers qualitative assessment and that the impact of rater experience can be lessened by training in qualitative protocol. However, determination of the sensitivity, specificity, positive and negative predictive value of quantitative vs. qualitative assessment of VFSS in dogs is lacking.

## Conclusions

The current peer-reviewed literature on fluoroscopic evaluation of the pharynx and UES in dogs shows a lack of standardization regarding imaging protocol and image interpretation across institutions. The most consistent feature identified is the acquisition of fluoroscopic studies at a rate of 30 frames per second. All institutions perform videofluoroscopy in right lateral or standing positions the impact of which is unknown for assessing pharyngeal collapse but which should have little baring on the outcome of quantitative swallowing parameters involving the pharynx and UES. Some institutions use qualitative measures to evaluate VFSS while others apply quantitative criteria. There does not appear to be a standard set of quantitative criteria applied amongst the institutions where they are used and there are significant differences in the outcomes of quantitative measures obtained from VFSS suggesting significant inter-observer or inter-institutional variability. A consensus statement regarding imaging protocol and what parameters should be used (sensitivity and specificity of qualitative vs. quantitative assessment) to interpret airway and swallowing videofluoroscopic studies of the pharynx and UES in dogs is needed along with targeted analysis of observer variability.

## Author Contributions

RP performed literature searches, reviewed the articles identified via literature search and determined which articles met inclusion criteria, and primarily authored the manuscript.

### Conflict of Interest Statement

The author declares that the research was conducted in the absence of any commercial or financial relationships that could be construed as a potential conflict of interest.
